# Expression of Cripto‐1 predicts poor prognosis in stage I non‐small cell lung cancer

**DOI:** 10.1111/jcmm.15518

**Published:** 2020-07-22

**Authors:** Chunhua Xu, Qi Yuan, Huidi Hu, Wei Wang, Qian Zhang, Li Li, Jiwang Wang, Rusong Yang

**Affiliations:** ^1^ Department of Respiratory Medicine The Affiliated Brain Hospital of Nanjing Medical University Nanjing China; ^2^ Clinical Center of Nanjing Respiratory Diseases and Imaging Nanjing China; ^3^ Department of Pathology The Affiliated Brain Hospital of Nanjing Medical University Nanjing China; ^4^ Department of Respiratory Medicine The First Affiliated Hospital of Nanjing Medical University Nanjing China; ^5^ Department of Thoracic Surgery The Affiliated Brain Hospital of Nanjing Medical University Nanjing China

**Keywords:** cripto‐1, metastasis, non‐small cell lung cancer, prognosis

## Abstract

Cripto‐1 (CR‐1) is related to the biological behaviour and prognosis of carcinomas. The purpose of this study was to investigate the significance of CR‐1 expression in surgically resected stage I non‐small cell lung cancer (NSCLC). One hundred and forty‐eight patients with completely resected stage I NSCLC and available clinical follow‐up data were assessed. The protein expression of CR‐1 in the tumours was detected by immunohistochemistry. CR‐1 was highly expressed in 64 of 148 tumours. Among patients with high CR‐1 expression, progression‐free survival and overall survival rate were significantly lower than those of patients with low CR‐1 levels (*P* = .013 and *P* = .019, respectively). The incidence of distant metastasis in patients with high CR‐1 expression was significantly higher than that of in patients with low CR‐1 expression (57.13% vs 21.43%, *P* = .001). The results of the multivariate analysis confirmed that a high CR‐1 was a significant factor for poor prognosis. In conclusion, CR‐1 could be a useful prognostic factor in patients with stage I NSCLC, likely as an indicator of the metastatic propensity of the tumour.

## 1 INTRODUCTION

Lung cancer is the most common cause of cancer‐related deaths worldwide.^1^ The tumour‐node‐metastasis (TNM) staging is an important determinant of prognosis in non‐small cell lung cancer (NSCLC). Nevertheless, as in many cases of malignant tumours, TNM staging based on tumours has proved limited to predict prognosis in individual. For example, the clinical courses of patients at the same stage show different form and trait, and 30% of NSCLC patients in stage I still die after complete resection, which is related to the fact that occult micrometastases may be present before surgery.^2^ Identifying new biomarkers that can predict postoperative recurrence risk of stage I NSCLC will be helpful in formulating post‐resection treatment strategies for high‐risk patients. Many possible biomarkers have been reported, such as p53, Ki‐67, and carcinoembryonic antigen (CEA), but none of them has gained routine clinical use in the postsurgical evaluation of these patients.^3^, ^4^, ^5^

Cripto‐1 (CR‐1) is a member of the epidermal growth factor‐cripto FRL1 cryptic family, which is overexpressed in various carcinomas.^6^, ^7^, ^8^ It has been reported that plasma CR‐1 might be a novel biomarker for the early detection of breast and colon carcinomas.^9^ CR‐1 is overexpressed in lung cancer tissues, and the level of CR‐1 expression is associated with the prognosis.^10^, ^11^ Recently, our studies showed that serum CR‐1 might be a potential biomarker for lung cancer diagnosis.^12^, ^13^ Until now, the expression of CR‐1 correlates with distant metastasis and predicts survival in stage I NSCLC patients has not been fully studied.

In the present study, the expression level of CR‐1 was tested by immunohistochemistry in completely resected stage I NSCLC patients to assess the relationship with prognosis. We found that the CR‐1 was overexpressed in lung cancer and that this overexpression was associated with a worse outcome for stage I NSCLC patients.

## 2 METHODS

### 2.1 Patients

The specimens were obtained from 148 cases of pathological stage I NSCLC. The patient stage was determined according to the eighth edition of TNM.^14^ The clinical information of patients was analysed. All patients received surgery alone and were followed up. Follow‐up was conducted every 3 months in the first postoperative year and every 6 months thenceforth. The following criteria were used to exclude patients: the patients received preoperative chemotherapy or radiotherapy, died within 3 months of surgery or succumbed to a cause other than NSCLC. Recurrence was determined by chest enhanced CT, whole‐body bone scan, head enhanced magnetic resonance imaging or positron emission tomography/computed tomography (PET/CT) and confirmed by definite pathological diagnosis when needed. After the follow‐up, the patients were classified as alive with disease, alive without disease and dying from lung cancer. No patient died of causes unrelated to cancer. Progression‐free survival (PFS) was calculated from the beginning date of surgery to any new recurrence. Overall survival (OS) was from the date of surgery to death from any cause or last follow‐up.

This study was approved by the ethics committee of the Affiliated Brain Hospital of Nanjing Medical University. All patients enrolled were provided with written informed consent.

### 2.2 Immunohistochemistry

Tissue microarrays (TMA) were constructed from formalin‐fixed paraffin‐embedded tissues with tumours. Three 4‐μm tissue cores from formalin‐fixed paraffin‐embedded donor blocks were precisely arrayed into a new recipient paraffin block. Each patient had three lung tumour tissue cores on a TMA slide. The immunohistochemical staining was used to detect the expression of CR‐1 protein in tumour tissues. 0.3% hydrogen peroxide reacted with methanol for 20 minutes to terminate the endogenous peroxidase activity. After washing with phosphate buffer saline, tissue sections were treated with 0.01‐mol/L citrate buffer (pH 6.0) and then irradiated in the microwave oven for 20 minutes. After irradiation, the slide was cooled at room temperature. Rabbit antibody specific to CR‐1 (1:100, monoclonal antibody 2771; Minneapolis R&D Systems Inc) was used as the first antibody. The sections were incubated overnight with the first antibody at 4°C, and then with the second antibody. Diaminobenzidine was used for result visualization. No primary antibody was detected by negative control staining in each group.

### 2.3 Immunohistochemical scoring

The immunohistochemical staining was scored by two pathologists. This is a single‐blind study, and two pathologists scored the slides independently. The expression level of each antibody in cancer cells was evaluated independently. The expression of CR‐1 was scored by assigning a percentage of positive cells and an intensity score. Immunohistochemical staining intensity was scored as 0, 1, 2 or 3, indicating absent, weak, moderate or strong expression, respectively. The percentages of positive cells were scored as follows: 0 < 1%, 1 = 1%‐33%, 2 = 33%‐67%, 3 = 67%‐100%. For each marker, the score of percentage and intensity was multiplied for final scores and to classify expression. The scores of 0‐3 were considered to be low level of expression, while the scores of more than 3 were considered high level of expression.^15^

### 2.4 ELISA for CR‐1

Serum samples were obtained from each patient after diagnosis confirmed. The supernatant was obtained by centrifuging the sample at −4°C for 10 minutes at 1500 *g*. CR‐1 levels were measured by ELISA kits (quantitative protein; R&D system) according to the manufacturer's instructions. All tests are in duplicate, diluted appropriately, and the technicians were blinded to clinical data.

### 2.5 Statistical analysis

The relationships between CR‐1 expression and clinicopathological parameters were analysed by Fisher's exact test or χ^2^ test as appropriate. The survival curves were estimated by the Kaplan‐Meier method, and the differences among them were compared by the log‐rank test. Cox's proportional hazards regression model was used to assess the impact of CR‐1 expression on PFS and OS after adjustment for tumour size, histological type and tumour grade. A value *P* < .05 was considered as significant.

## 3 RESULTS

### 3.1 Clinical characteristics

The patient's characteristics were shown in Table 1. The mean age of the patients enrolled was 63.5 years. The proportion of males and females accounted for 47.3% and 52.7% of lung cancer patients, respectively. In postoperative pathology, 40 cases were squamous cell carcinomas and 108 cases were adenocarcinomas. Fifty‐four (36.49%) cases were differentiated well (G1), 52 (35.14%) cases were differentiated moderately (G2), and 42 (28.38%) cases were differentiated poor (G3). One hundred and two patients were at stage T1, and 46 patients were at stage T2. After a 5‐year follow‐up, the patient's data were analysed. The median OS was 30 months, and the median PFS was 21 months.

**TABLE 1:** Clinicopathological characteristics of NSCLC patients

Characteristic	N	%
Age (y)		
Range	35‐76	
Mean	58.5	
Gender		
Male	70	47.3
Female	78	52.7
Smoking condition		
Non‐smoker	100	67.6
Smoker	48	32.4
Histological type		
SCC	40	27.0
ADC	108	73.0
Tumour differentiation		
Well	52	35.1
Moderate	54	36.5
Poor	42	28.4
Tumour size		
T1	102	68.9
T2	46	31.1

Abbreviations: ADC, adenocarcinoma; SCC, squamous cell carcinoma.

### 3.2 CR‐1 expression in stage I NSCLC

**FIGURE 1:**
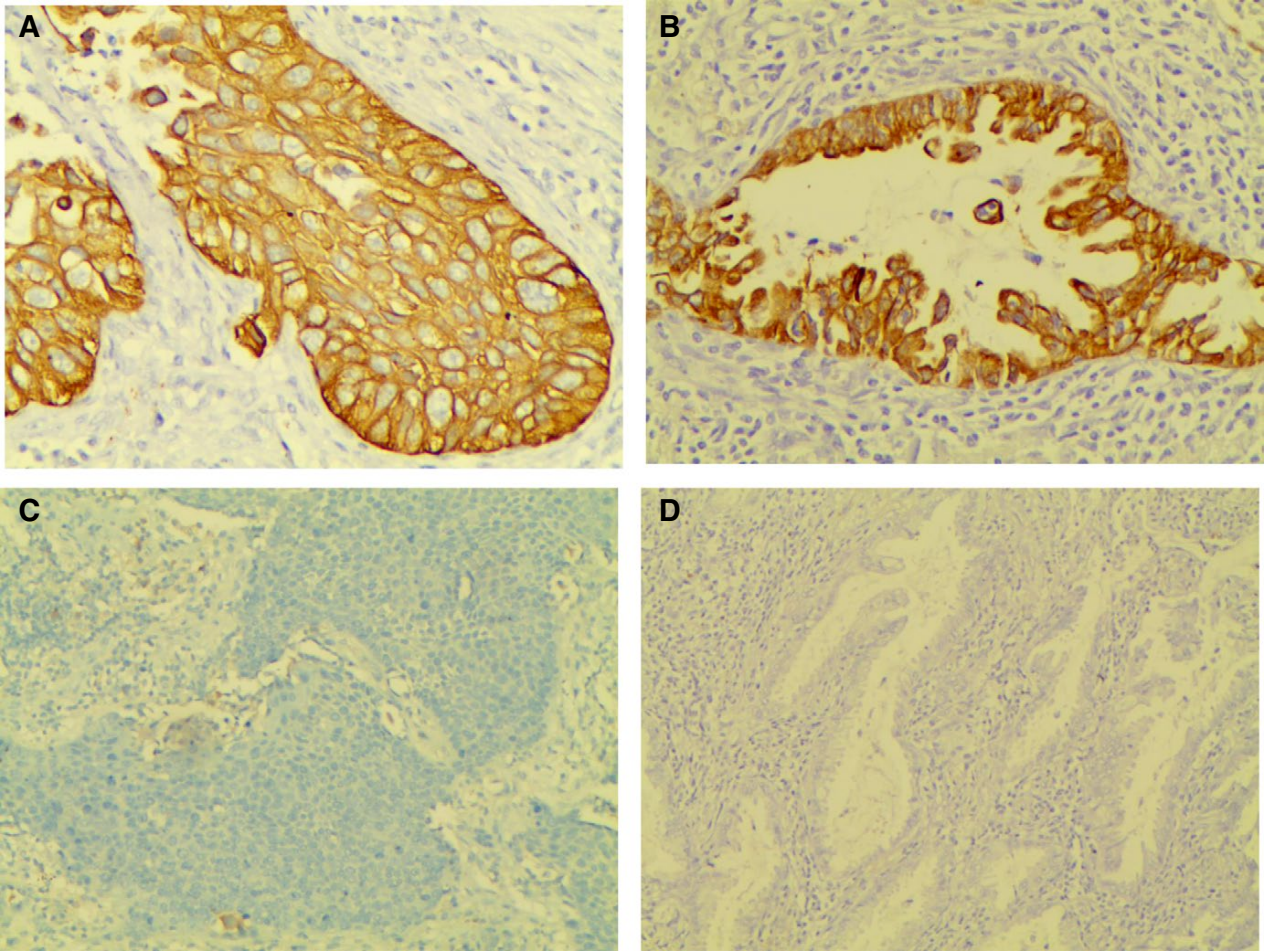
Immunohistochemical analysis of CR‐1 in NSCLC patients. A strong cytoplasmic reactivity is evident in a squamous cell carcinoma (A) and in an adenocarcinoma (B). Negative immunostaining of a squamous cell carcinoma (C) and an adenocarcinoma (D) (original magnification × 400)

The specific CR‐1 staining was mainly localized in the cytoplasm. Besides, positive staining was also detected in the nuclei of tumour cells. Based on the CR‐1 immunoreactivity score, 64 of 148 patients had high CR‐1 expression and 84 patients had low CR‐1 expression. The examples of CR‐1 immunostaining in different histotypes are shown in Figure 1.

Table 2 presents the relationships between CR‐1 expression and clinicopathological characteristics of NSCLC patients. We found that the expression level of CR‐1 was correlated with tumour differentiation and tumour size significantly (*P* = .043 and .033). Meanwhile, statistical analysis showed no correlations between CR‐1 expression and age, gender, histological type and smoking condition.

**TABLE 2:** CR‐1 status in stage I NSCLC tumours according to the clinicopathological features of patients

Variable	Low CR‐1 expression (N)	High CR‐1 expression (N)	*P*
Age (y)			
<60	36	26	.867
≥60	48	38	
Gender			
Male	40	30	.928
Female	44	34	
Smoking condition			
Non‐smoker	58	42	.724
Smoker	26	22	
Histological type			
SCC	24	16	.628
ADC	60	48	
Tumour differentiation			
Well‐moderate	66	40	.043
Poor	18	24	
Tumour size			
T1	64	38	.033
T2	20	26	

Abbreviations: ADC, adenocarcinoma; SCC, squamous cell carcinoma.

**FIGURE 2:**
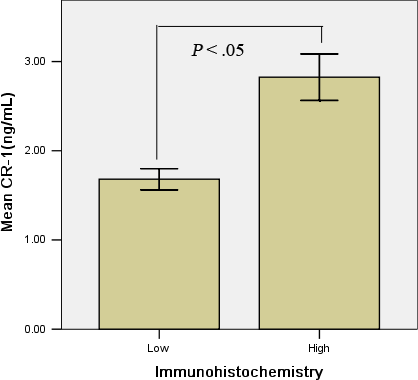
Association between CR‐1 expression evaluated immunohistochemically and protein levels determined by ELISA. Fifty‐four randomly selected tumours were analysed by immunohistochemistry and regarded as high or low. The same tumours were assayed for CR‐1 levels by ELISA. The graph shows the quantitative CR‐1 levels of high and low samples. Also indicated are means; bars, ±SD. The difference was statistically significant (*P* = .017)

ELISA of CR‐1 was performed on the serum to verify the correlation between quantitative determination of protein level and immunohistochemical expression. These samples showed different levels of protein expression in immunohistochemistry. We found an association between the immunohistochemical evaluation of CR‐1 expression and the levels of the protein determined by ELISA (Figure 2). The average content of CR‐1 expression was (1.39 ± 0.87) ng/mL of tumours for low and (2.82 ± 1.73) ng/mL for high, which were statistically significant different (*P* = .017).

### 3.3 CR‐1 expression and distant metastasis

In the study population, 54 (36.49%) patients developed distant metastasis at different time intervals after surgery, which was associated with the level of CR‐1 expression in tumour. Distant metastasis occurred in 21.43% (18 of 84) patients with low CR‐1 expression, nevertheless, the proportion increased to 53.13% (34 of 64) in the high CR‐1 expression ones (*P* = .001).

### 3.4 CR‐1 expression and postoperative survival

The mean follow‐up for PFS was 54 months for those who were alive and remained relapse‐free at last follow‐up. The mean follow‐up for OS for those who remained alive at last follow‐up was 58 months. The Kaplan‐Meier plots showed that the 5‐year PFS rates and the 5‐year OS rates were 55.41% and 59.46% of all patients, respectively.

**FIGURE 3:**
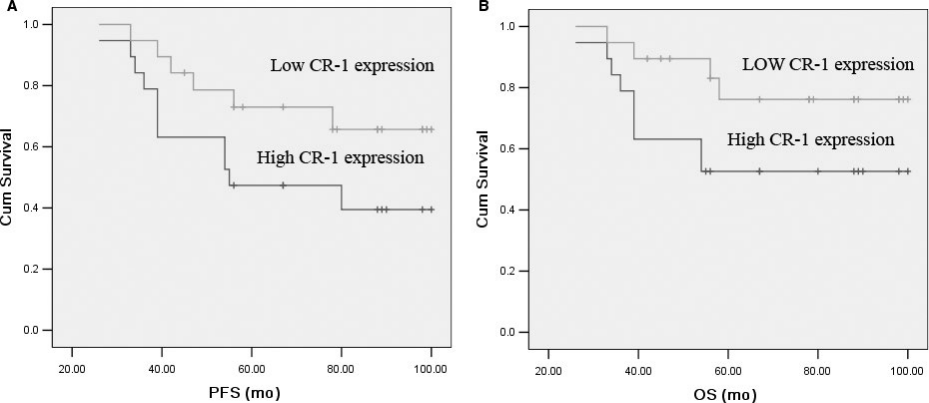
Progression‐free (A) and overall survival (B) in the 148 NSCLC patients based on high or low CR‐1 expression. Log‐rank test determined that the PFS (A) and OS (B) in high CR‐1 patients were significantly shorter than those in the low CR‐1 patients (*P* = .002, *P* = .001)

The postoperative survival curves showed significantly poorer prognosis for patients with high CR‐1 expression levels. The 5‐year PFS rates of patients with high and low CR‐1 expression were 31.25% and 73.81%, respectively (*P* = .013). CR‐1 proved to affect OS as well, with the 5‐year survival rates were 35.94% for the high CR‐1 expression and 77.38% for the low CR‐1 expression (*P* = .019, Figure 3).

There was no significant correlation between PFS or OS and tumour size, histological type and differentiation. Multivariate analysis confirmed that high CR‐1 expression was independent factor for poor prognosis for both PFS (*P* = .031) and OS (*P* = .028, Table 3).

**TABLE 3:** Multivariate analysis of prognostic variables for PFS and OS

Variable	PFS		OS	
	HR (95% CI)	*P*	HR (95% CI)	*P*
Age (<60 vs ≥60)	0.671 (0.346‐1.320)	.0238	0.690 (0.245‐1.943)	.483
Gender (Male vs Female)	1.667 (0.848‐3.278)	.0139	1.870 (0.775‐4.516	.164
Histological type (SCC vs ADC)	0.927 (0.454‐1.892)	.0835	0.689 (0.351‐1.353)	.279
Differentiation (Well‐moderate vs Poor)	1.053 (0.375‐2.950)	.0922	1.943 (0.605‐6.240)	.265
Tumour size (T1 vs T2)	1.134 (0.483‐2.66)	.773	1.706 (0.439‐6.630)	.441
CR‐1 (Low vs High)	1.832 (1.055‐3.179)	.00031	1.734 (1.383‐2.665)	.023

Abbreviations: ADC, adenocarcinoma; CI, confidence interval; HR, Hodds ratio; OS, overall survival; PFS, progression‐free survival; SCC, squamous cell carcinoma; SE, standard error.

## 4 DISCUSSION

In the present study, the expression of CR‐1 protein was analysed by immunohistochemistry in 148 patients with stage I NSCLC. The serum levels of CR‐1 have been proved prognostic value in several tumours, such as colon cancer and lung cancer.^9^, ^12^

Here, we showed that CR‐1 overexpression was detected in 64 out of 148 patients with stage I NSCLC. When the immunohistochemical patterns of expression were analysed comparatively with the follow‐up data, they stratified the patients into markedly different survival groups, patients with high CR‐1 expression in the tumour had a significantly shorter overall and disease‐free survival times than those with low CR‐1 expression. Moreover, the expression of CR‐1 resulted to be significantly associated with the establishment of distant metastasis. There was correlation found between the CR‐1 and tumour size and tumour differentiation, thus supporting the contention that the prognostic significance of CR‐1 expression is consistent with that of these conventional prognostic indicators.

The prognostic value of CR‐1 may be due to its correlation with distant metastasis. It has been reported that there is a positive correlation between high levels of CR‐1 in serum and metastasis development in lung cancer patients.^13^ CR‐1 plays an important role in tumorigenesis by promoting cell proliferation, survival, migration and invasion, inducing epithelial to mesenchymal transition, transformation, branching morphogenesis and tumour angiogenesis.^16^ However, the mechanism of the role of CR‐1 in the tumours metastasis remains unknown. CR‐1 not only acts as a nodal co‐receptor, but also mediates signal transduction of other transforming growth factor‐beta ligands.^17^ Conversely, binding of CR‐1 to transforming growth factor‐beta (TGF‐β) and activin inhibits the signal transduction of TGF‐β and activin mammalian cells.^18^ In addition, CR‐1 can activate PI3‐K/AKT/GSK‐3β and ras/raf/MAPK signalling pathways.^6^ It has been reported that CR‐1 promotes the expression of markers and signalling molecules associated with epithelial‐mesenchymal transition.^19^

The major histological types of lung cancer are associated with smoking, although the association is greater for squamous cell carcinoma than for adenocarcinoma. Adenocarcinoma is the most common form of lung cancer in never smokers.^20^ The specific molecular and pathological features might be associated with lung adenocarcinomas arising in non‐smoker.^21^ In the present study, the majority of male patients with smoking were mainly squamous cell carcinoma, suggesting that smoking is closely related to the squamous cell carcinoma occurrence. The majority of female patients with non‐smoking are adenocarcinoma, suggesting that female has relatively independent risk factors, which is similar to previous reports.^22^

Several prognostic indicators of stage I NSCLC have been reported, some of which, for example the angiogenesis and the interleukin‐2 receptor, are considered as biomarkers for metastasis tendency.^23^, ^24^, ^25^, ^26^ We believe that, as pointed out in literature, in order to completely understand the prognostic value of biomarkers, it is necessary to assess the interrelationship between them to conduct a comprehensive prognostic index integrating different biomarkers. The results of the study demonstrate that CR‐1 protein can be regarded as one of the biomarkers inclusion in the development of a prognostic model for stage I NSCLC, and may be an indicator of the metastatic propensity.

Several limitations of our study warrant discussion. First, we performed the study at a single centre with relatively small sample size. Second, the expression of CR‐1 was detected by immunohistochemistry, not by quantitative real‐time reverse transcription polymer chain reaction and Western blots. Third, the specific underlying mechanism of the relationship between CR‐1 expression and NSCLC distant metastasis and prognosis was lacking.

In conclusion, CR‐1 is a prognostic factor in stage I NSCLC patients, and may be related to lung cancer distant metastasis.

## CONFLICT OF INTEREST

The authors declare no any conflicts of interest in this work.

## AUTHOR CONTRIBUTIONS

**Chunhua Xu:** Conceptualization (equal); Funding acquisition (equal); Writing‐original draft (equal); Writing‐review & editing (equal). **Wei Wang:** Supervision (equal); Writing‐review & editing (supporting). **Qian Zhang:** Data curation (equal); Investigation (equal); Software (equal). **Li Li:** Investigation (supporting); Visualization (equal). **Rusong Yang:** Project administration (equal); Resources (equal). **Jiwang Wang:** Conceptualization (supporting). **Huidi Hu:** Formal analysis (equal); Methodology (equal). **Qi Yuan:** Data curation (equal); Formal analysis (equal); Supervision (equal).

## Data Availability

Data sharing is not applicable to this article as no new data were created or analysed in this study.
